# Multi-view affinity-based projection alignment for unsupervised domain adaptation via locality preserving optimization

**DOI:** 10.1038/s41598-025-05331-3

**Published:** 2025-07-01

**Authors:** Weibin Luo, Mingye Chen, Jian Gao, Yanping Zhu, Fang Wang, Chenyang Zhu

**Affiliations:** 1 School of Computer Science and Artificial Intelligence, Changzhou University, Changzhou, 213000 China; 2 Department of Computer Science, Brunel University London, London, UB8 3PH UK

**Keywords:** Unsupervised domain adaptation, Multi-view learning, Locality preserving projection, Feature alignment, Pseudo-labeling, Vision transformer, Computational science, Computer science, Information technology, Software

## Abstract

Unsupervised Domain Adaptation (UDA) aims to transfer knowledge from a labeled source domain to an unlabeled target domain with differing data distributions. However, it remains difficult due to noisy pseudo-labels in the target domain, inadequate modeling of local geometric structure, and reliance on a single input view that limits representational diversity in challenging tasks. We propose a framework named Multi-view Affinity-based Projection Alignment (MAPA) that uses a teacher–student network and multi-view augmentation to stabilize pseudo-labels and enhance feature diversity. MAPA transforms each sample into multiple augmented views, constructs a unified affinity matrix that combines semantic cues from pseudo-labels with feature-based distances, and then learns a locality-preserving projection to align source and target data in a shared low-dimensional space. An iterative strategy refines pseudo-labels by discarding low-confidence samples, thereby raising label quality and strengthening supervision for the target domain. MAPA also employs a consistency-weighted fusion mechanism to merge predictions from multiple views, improving stability under domain shift. Finally, MAPA leverages class-centric and cluster-level relationships in the projected space to further refine label assignments, enhancing the overall adaptation process. Experimental results on Office-Home, ImageCLEF, and VisDA-2017 show that MAPA surpasses recent state-of-the-art methods, and it maintains robust performance across backbones including ResNet-50, ResNet-101, and Vision Transformer (ViT).

## Introduction

Deep learning has achieved remarkable success in a wide range of computer vision tasks, such as cross-domain object detection, adaptive visual quality assessment, and label-efficient semantic segmentation ^1–3^. Despite these successes, deep learning models often suffer performance degradation when applied to data distributions that differ from those seen during training. This issue is particularly pronounced in real-world applications, where the domain discrepancy may stem from changes in lighting, background, sensor type, or acquisition protocol. Unsupervised Domain Adaptation (UDA) focuses on transferring knowledge from a labeled source domain to an unlabeled target domain, where both domains share the same label space but differ in data distribution. The mismatch between the domains can degrade the model’s generalization performance when it is applied directly to the target domain. UDA has become widely studied for applications where collecting labels in the target domain is expensive or impractical. Examples include autonomous driving in diverse cities and weather conditions ^4^, medical image analysis across multiple scanners or institutions ^5^, and lithology classification in geophysical exploration through adversarial UDA strategies ^6^. By reducing the distribution difference while retaining task-relevant semantics, UDA allows models to generalize to unseen domains and has grown into an important topic in computer vision and machine learning.

Although UDA methods have advanced significantly in recent years, several important difficulties remain. One key challenge is the reliance on pseudo-labels in the target domain, which are often noisy in the early stages and can misdirect the model ^7^. Another issue is that many methods concentrate only on global distribution alignment, overlooking the local geometric structure of data that is crucial for maintaining semantic consistency. In addition, most UDA frameworks employ only a single view of input images, reducing their ability to learn robust and varied representations. These factors limit the effectiveness of adaptation in complex tasks, especially when confronted with large domain shifts or cross-modal conditions.

To overcome these challenges, we propose a framework called Multi-view Affinity-based Projection Alignment (MAPA). The method has two main phases. In the first phase, we extract contextual features with a teacher–student network, where the student is trained using source labels and pseudo-labeled target data, and the teacher is updated by a moving average scheme to maintain stable pseudo-labels. Each input image is transformed into three augmented views: Gaussian blur with color variation, random erasing with color variation, and grayscale conversion with color variation. These views broaden the representation space by offering additional viewpoints. In the second phase, MAPA constructs a joint affinity matrix by integrating semantic similarity from pseudo-labels and structural similarity from feature distances. This affinity matrix drives a locality-preserving projection that aligns the source and target domains in a common low-dimensional space while keeping the local structure intact. Within this space, we measure distances from target samples to source class centroids and clusters in the target domain, forming two probability distributions. These distributions are combined in each view to create view-specific predictions, which are then merged through a consistency-weighted approach. This produces final pseudo-label distributions, and high-confidence labels are chosen for the next training cycle. The procedure is repeated to refine the subspace and label accuracy. We validate MAPA on three standard UDA benchmarks, namely Office-Home, ImageCLEF, and VisDA-2017, by using multiple backbone networks such as ResNet-50, ResNet-101, and Vision Transformer (ViT). Our method outperforms recent state-of-the-art approaches across all benchmarks and yields strong performance even in the presence of large domain shifts. Furthermore, ablation studies confirm the individual and joint benefits of multi-view learning and subspace alignment.

The core contributions of this work are the introduction of a unified approach for unsupervised domain adaptation that tackles several limitations of current methods, including sensitivity to noisy pseudo-labels, the challenge of capturing local geometric structure, and restricted variety in feature representation. The specific contributions are as follows:

- We present MAPA, a unified domain adaptation framework that integrates multi-view feature augmentation and projection-based subspace alignment. MAPA uses three complementary augmentation views to capture diverse and robust representations. A consistency-aware fusion approach merges view-specific predictions, improving stability under domain shift.
- We propose a new affinity matrix construction technique that combines semantic consistency from pseudo-labels and structural similarity derived from feature distances. This unified affinity matrix supports the learning of a locality-preserving projection to align source and target data in a common low-dimensional space. Within this space, class-level and cluster-level relationships are iteratively used to refine pseudo-labels, which raises the quality of target supervision.
- We conduct thorough evaluations on three benchmark datasets using multiple backbone networks, including ResNet-50, ResNet-101, and ViT. MAPA consistently surpasses state-of-the-art approaches across all scenarios. In addition, comprehensive ablation studies confirm the effectiveness and complementary nature of the proposed modules.

The rest of the paper is organized as follows. “Related work” discusses recent work in unsupervised domain adaptation, including label-centered techniques, feature-label fusion, and regularization strategies. “Proposed methodology” describes the MAPA framework, covering its teacher-student feature extraction module, multi-view augmentation, and projection alignment. “Experimental results and analysis” presents the experimental setup and provides detailed evaluations on multiple benchmarks and backbone networks, including ablation studies and visualization analyses to confirm the value of each component. Finally, “Conclusion and future work” summarizes the main findings and considers possible future research directions.

## Related work

UDA has attracted considerable attention in computer vision and geoscience applications, particularly due to the scarcity of labeled target data in real-world scenarios. Existing methods vary in terms of how they handle pseudo-labels, feature alignment, regularization, and model robustness.

### Pseudo-labeling and confidence refinement

Pseudo-label generation and confidence-based sample selection play central roles in methods that aim to establish reliable supervisory signals from unlabeled data. Structured Prediction for Selective Pseudo-Labeling (SPL) ^8^ employs a clustering process to generate pseudo-labels, progressively including the most trustworthy samples for training. Masked Image Consistency (MIC) ^9^ introduces random patch masking and a consistency loss to leverage spatial context for more stable adaptation. Progressive Pseudo Pair Generation (P3G) ^10^ iteratively synthesizes self-supervised signals by producing pseudo sharp-blurry image pairs, inspired by CycleGAN’s cyclic consistency principle, tailored for blur degradation alignment.Several recent semi-supervised domain adaptation (SSDA) methods have contributed valuable insights into pseudo-label optimization under limited supervision. Ngo et al. ^11^ proposed a Trico-training framework that co-trains an MLP with two GCN classifiers to improve pseudo-label quality by modeling inter- and intra-domain relationships through structure-aware disagreement. Similarly, Kim et al. ^12^ introduced DARK, which distills domain-specific knowledge across augmented views and applies sample-wise dynamic weighting to stabilize pseudo-label refinement. Although these methods operate under SSDA assumptions, their strategies for enhancing pseudo-label reliability and leveraging multi-view consistency offer useful references for fully unsupervised adaptation.

Cross-Modal Knowledge Distillation (CMKD) ^13^ transfers knowledge from vision-language models, including CLIP ^14^ and UniMoS ^15^, to facilitate adaptation on unlabeled target data. Some studies incorporate high-confidence sample selection to guide training, as demonstrated in Domain Adaptation via Prompt Learning (DAPL) ^16^, which gradually integrates target instances that best match the source distribution. The complexity of data annotation in real-world tasks such as blind image quality assessment (BIQA) is also highlighted in distortion-guided unsupervised domain adaptation for BIQA (DGQA) ^17^, emphasizing the additional burdens that arise when domain shifts occur in authentic image datasets. Moreover, ExMap ^18^ applies a clustering module to generate pseudo-labels from explanatory heatmaps, replacing ground truth labels to promote more flexible training.

### Adversarial and transformer-driven alignment

There is also research focusing on adversarial frameworks and transformer architectures to align feature distributions across domains. Conditional Adversarial Domain Adaptation (CDAN) ^19^ extends adversarial training by integrating classifier predictions with feature embeddings through multilinear operations, enabling more discriminative alignment. Backprop Induced Feature Weighting for Adversarial Domain Adaptation (BIWAA) ^20^ generates feature weight vectors via classification loss backpropagation, guiding adversarial networks to concentrate on informative features. Cross-Domain Gradient Discrepancy Minimization (CGDM) ^21^ aligns gradients between source and target samples and employs a clustering-based pseudo-labeling strategy to refine adaptation.

Recent advances in transformer models have further enriched UDA research. Contrastive Vicinal Space for Unsupervised Domain Adaptation (CoVi) ^22^ proposes EMP-Mixup, an entropy-based approach to locate vicinal points between source and target domains, dividing the resulting space for both contrastive and consensus-based alignment. Patch-Mix Transformer (PMTrans) ^23^ constructs an intermediate domain by mixing patches from source and target images, formulating a min-max cross-entropy objective for improved domain transfer. Transferable Vision Transformer (TVT) ^24^ introduces a Transferability Adaptation Module and a Discriminative Clustering Module to enhance feature alignment using ViTs. The Explicit Class Boundaries (ECB) method ^25^ jointly optimizes ViTs and Convolutional Neural Networks (CNNs) for precise category boundary detection. Meanwhile, MLRGL ^26^ incorporates affinity-based propagation with low-rank constraints and multiview feature integration, further broadening the scope of transformer-era alignment techniques.

### Diffusion-based representation alignment

Recent progress in generative modeling has promoted the use of diffusion models (DMs) in UDA, especially for challenging scenarios such as adverse weather, medical imaging, and sensor-based applications. Compared to GANs, DMs offer more stable training and superior fidelity, making them ideal for generating high-quality target-style data. Shen et al. ^27^ proposed ControlUDA, which leverages pre-trained text-to-image diffusion models to generate target-like images for segmentation under adverse weather, enhanced by UDAControlNet for prompt-based generation and label filtering. ControlUDA achieves 72.8% mIoU on Cityscapes-to-ACDC.

Zhao et al. ^28^ introduced Diffusion-UDA for fault diagnosis in submersible systems, using diffusion models with contrastive learning to bridge signal distribution gaps across components. In addition, Zeng et al. ^29^ presented Diff-Unmix, a self-supervised framework for hyperspectral image (HSI) denoising, combining transformer-based spectral unmixing with conditional diffusion to reconstruct noise-free HSI representations.

DiffusionGAN3D ^30^ integrates 3D GANs with diffusion priors for text-guided 3D generation and domain adaptation, enabling controllable and high-quality 3D synthesis. Benjilali et al. ^31^ proposed DATUM, a one-shot UDA method that uses Stable Diffusion to synthesize semantically guided, diverse target-like images from a single unlabeled sample. DATUM surpasses previous OSUDA methods by up to 7.1%, showing the potential of diffusion models in low-resource settings.

### Multi-source domain adaptation strategies

While most UDA methods assume a single labeled source domain, recent studies have explored multi-source unsupervised domain adaptation (MSUDA) to better address the diversity and coverage of real-world target domains. Ngo et al. ^32^ proposed a divide-and-conquer MSUDA framework that decomposes the adaptation problem into multiple single-source sub-tasks and solves each using a task-specific model. These models are trained collaboratively, which helps mitigate negative transfer caused by dominant source bias and enhances representation robustness.

In the context of semantic segmentation, Park et al. ^33^ introduced a pseudo-label rectification framework that leverages co-teaching and pseudo-label decoupling across multiple source models. Their method updates peer networks using non-integrated pseudo labels and refines predictions only when model disagreements arise, which improves label quality and class balance, particularly for small or underrepresented regions. In addition to natural image datasets, multi-source UDA has been actively explored in remote sensing (RS) applications, where labeled data are especially scarce. Ngo et al. ^34^ proposed MECKA, a multi-expert collaboration framework that integrates knowledge from heterogeneous remote sensing sources. The method first constructs view-specific representations to preserve the semantic characteristics of each source domain, then connects these views through collaborative learning to leverage their complementary strengths. Unlike approaches that naively merge all sources into one, MECKA emphasizes maintaining inter-source diversity, which is crucial when class coverage is imbalanced or incomplete. Experiments on RS scene classification benchmarks demonstrate its superior performance under both complete and incomplete MSUDA settings.

Although these MSUDA methods demonstrate strong performance by leveraging inter-source diversity, our approach targets a complementary single-source scenario. Rather than relying on multiple labeled domains, we construct multiple augmented views from a single source and align them with the target domain through affinity-based projection and confidence-aware pseudo-labeling. This design promotes robust domain adaptation even in resource-constrained settings where only one source domain is available.

### Feature representation and regularization techniques

Another category of UDA explores parameterization and regularization strategies to promote domain-invariant representations while retaining domain-specific details. Adversarial Spectral Adaptation Network (ASAN) ^35^ enforces spectral alignment to preserve domain structures during feature extraction. Prompt Gradient Alignment (PGA) ^36^ adopts a multi-objective formulation by unifying gradient alignment with norm penalization to improve generalization under challenging shifts. Gradual Source Domain Expansion (GSDE) ^37^ systematically incorporates high-confidence target instances as pseudo-source data, refining adaptation in structured stages. Environment Label Smoothing (ELS) ^38^ stabilizes training with softened labels, mitigating the impact of noisy environment annotations.

Smooth Domain Adversarial Training (SDAT) ^39^ studies the smoothness of the loss landscape, selectively enhancing the smoothness of the classification component for robust adaptation. Global-Local Optimal Transport based Distributional Robustness (GLOT-DR) ^40^ merges local and global regularization to fortify UDA and semi-supervised learning. Margin-based uncertainty measures from ^41^ sample differentially near decision boundaries of occupancy functions, improving model reliability when labels are unavailable. Additionally, the Dynamic Kernel Prior (DKP) method ^42^ estimates unknown super-resolution degradation kernels via a synergistic parameterized and regularization-based design, aiding blind super-resolution across domains.

Despite these methodological advancements, several issues persist. First, heavy reliance on pseudo-labels can introduce significant label noise, triggering error accumulation as training proceeds. Second, methods that combine feature and label information may lack a flexible weighting scheme, limiting their capacity to adapt to heterogeneous data distributions. Third, capturing intricate relationships in geoscience or computer vision tasks can be difficult, especially when the underlying data exhibits high nonlinearity or complex degradation processes. These challenges are accentuated by the labor-intensive nature of data annotation in tasks such as blind image quality assessment ^17^.

To address these gaps, the proposed MAPA mitigates pseudo-label noise through a teacher-student network, which uses a moving average update scheme to stabilize pseudo-labels and iteratively selects high-confidence samples. It applies a consistency-weighted strategy to integrate multiple augmented views, enabling more flexible adaptation to heterogeneous data distributions. Additionally, a joint affinity matrix fuses semantic consistency and structural similarity to guide a locality-preserving projection, capturing complex feature relationships under large domain shifts.

## Proposed methodology

### Unsupervised domain adaptation formulation

**Table 1:** Summary of notations used in the proposed method.

Symbol	Description
$x_i^s, y_i^s$	Source sample and its one-hot label
$x^t$	Target domain sample input
$r_{\theta }(x)$	Predicted class probabilities from student network
$r_{\theta }(x)_j$	Response (confidence) for class *j*
$\mathscr {U}$	Relational matrix encoding inter-class similarity
$f_i^s, g_i^s$	Feature representations of source sample $x_i^s$
$f_j^t, g_j^t$	Feature representations of target sample $x_j^t$
$d_{\epsilon }$	Domain discriminator with parameters $\epsilon$
$f \oplus g$	Concatenated features for domain discrimination
$\lambda _{da}, \lambda _{adv}$	Weights for domain alignment and adversarial loss
$\varphi , \theta$	Parameters of teacher and student networks
*X*	Concatenated feature matrix from source and target domains
$X_S$, $X_T$	Source and target domain feature matrices
$x_i$	A single feature vector from *X*
$\mu$	Mean vector of *X*
$P_{\text {pca}}$	PCA projection matrix
$X_{\text {reduced}}$	Dimensionality-reduced features after PCA
$\tilde{x}_i$	L2-normalized feature vector
$W_{\text {all}}$	Affinity matrix combining label and feature similarity
$\alpha$	Weighting coefficient for label similarity
$\sigma$	Gaussian kernel bandwidth parameter
*L*	Graph Laplacian matrix
*D*	Diagonal degree matrix of $W_{\text {all}}$
*P*	Projection matrix learned via LPP
$\tilde{X}^s_{\text {proj}}, \tilde{X}^t_{\text {proj}}$	Projected and normalized source and target features
$\mu _S, \mu _T$	Source class mean and target cluster mean in projected space
$\tau _T(i)$	Confidence score for target sample *i*
$\theta _{c(i)}$	Class-specific pseudo-label confidence threshold
*p*	Dynamic selection ratio decreasing over iterations
$\text {prob}(i)$	Max predicted class probability for sample *i*
$\text {pseudoLabels}_T(i)$	Refined pseudo-label for sample *i*
$\textbf{P}_v$	Predicted probability matrix from view *v*
$\alpha _v$	Weight for view *v* in fusion
$D_v$	Distance-based score for view *v*
$\gamma _v$	Bandwidth parameter for view weight computation
$\textbf{P}_{\text {fused}}$	Fused probability matrix from all views
$\hat{y}_T(i)$	Final predicted label for target sample *i*

We consider the UDA problem within the context of a *C*-class classification task. Let the input feature space be a *d*-dimensional Euclidean space, denoted as $\mathscr {X} = \mathbb {R}^d$, and the label space be $\mathscr {Y} = \{1, 2, \dots , C\}$. The source domain is defined as $D^s = \{(x_j^s, y_j^s)\}_{j=1}^m$, consisting of *m* labeled samples, where $x_j^s \in \mathscr {X}$ and $y_j^s \in \mathscr {Y}$. The target domain is defined as $D^t = \{x_j^t\}_{j=1}^n$, comprising *n* unlabeled samples with $x_j^t \in \mathscr {X}$.

Although the source and target domains exhibit distributional differences, they share the same label space $\mathscr {Y}$. The objective of UDA is to learn a classification model using both $D^s$ and $D^t$, such that it performs well on the target domain, despite the absence of target labels during training.

To address the domain discrepancy, we adopt the Locality Preserving Projection (LPP) technique, which aims to project both source and target data into a shared low-dimensional subspace. LPP preserves the local structure of the data while facilitating alignment between domains, thereby reducing the domain shift. The central challenge lies in constructing a feature transformation and classification model that maintains discriminative power in the source domain and generalizes effectively to the target domain, under the constraint of differing marginal distributions.

For clarity, we summarize the key notations used throughout the UDA formulation and our proposed method in Table 1. These include the main variables for domain definition, model parameters, and loss functions that will be referenced in subsequent sections.

### Overview of the MAPA framework

**Fig. 1:**
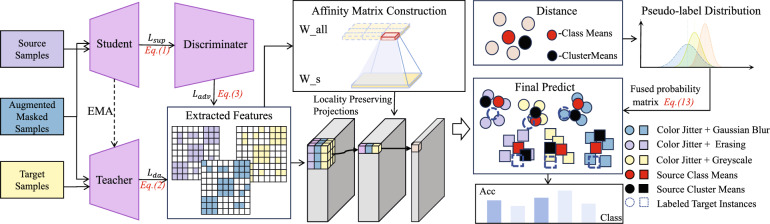
MAPA consists of multi-view feature extraction, affinity construction, projection alignment, and final pseudo-label generation.

The architecture of the proposed MAPA method is depicted in Fig. 1. MAPA operates in two main stages: (1) multi-view feature extraction through a teacher–student paradigm, and (2) affinity matrix guided projection alignment combined with iterative pseudo-label refinement.

In the feature extraction stage, the student network processes both labeled source domain samples and augmented versions of unlabeled target domain samples. Simultaneously, a teacher network, updated using exponential moving average (EMA) ^43^ of the student’s parameters, produces pseudo-labels for the target samples. A domain discriminator is jointly trained with the student network using adversarial loss to reduce domain-specific discrepancies and enforce feature invariance. To enhance feature diversity and improve robustness, each target sample is augmented into three distinct views through stochastic transformations: (i) color jittering combined with Gaussian blur, (ii) random erasing, and (iii) grayscale conversion.

Following feature extraction, MAPA constructs two complementary affinity matrices: one derived from pseudo-label similarities, and the other from pairwise feature distances computed using a Gaussian kernel. These matrices are integrated into a unified affinity matrix $W_{\text {all}}$, which serves as the input for LPP. The LPP algorithm projects the high-dimensional features into a common subspace that preserves local neighborhood structures while enhancing cross-domain alignment.

Within this shared subspace, distances between projected target features and both the source class centroids and target cluster centroids are measured to estimate soft pseudo-label distributions. Each augmented view contributes an individual prediction distribution. These distributions are integrated using a consistency-aware weighting strategy that emphasizes agreement among views, resulting in a refined pseudo-label probability matrix. High-confidence target samples are then selected based on this matrix to update pseudo-labels. This process is iteratively performed, progressively improving the quality of pseudo-labels and reducing domain shift.

By combining semantic information and local structural cues from multiple views, MAPA effectively addresses the challenges posed by complex and non-linear domain shifts, enabling more reliable unsupervised domain adaptation in visual recognition tasks.

### Domain-invariant feature learning

In this work, we address UDA by learning domain-invariant feature representations common to both source and target domains. The proposed method consists of three primary components: a **teacher network**, a **student network**, and a **discriminator**. The teacher network progressively refines its parameters by integrating previously learned representations with the latest updates from the student network. To achieve a stable learning process, the teacher network adopts a moving-average update strategy, combining newly updated parameters from the student network with historically accumulated information. This gradual parameter update scheme helps ensure that the generated pseudo-labels remain consistent and reliable, preventing large fluctuations during model training.

The balance between recent updates and accumulated historical knowledge is regulated by a smoothing coefficient. A higher value for this coefficient enhances stability but results in slower adaptation to new information, whereas a lower value allows the network to adapt more rapidly but increases the risk of instability due to noisy updates. Through this continuous refinement process, the teacher network ensures consistent pseudo-labels, significantly improving alignment between source and target domain representations.

The student network is trained using supervised loss computed from labeled samples within the source domain, as described by the cross-entropy loss in Eq. (1), following the domain adaptation principles outlined in EMA:

1 $$\begin{aligned} \mathscr {L}_{sup}(x^s, y^s) = -\sum _{i=1}^{C} y_i^s \log (r_{\theta }(x_i^s)), \end{aligned}$$

where $x_i^s$ denotes the $i$-th sample from the source domain, $y_i^s \in \{0,1\}^C$ is the corresponding one-hot encoded ground-truth label, and $r_{\theta }(x_i^s) \in \mathbb {R}^C$ is the predicted class probability vector produced by the student network with parameters $\theta$. This supervised objective encourages the model to correctly classify labeled source samples.

To enhance the discriminability of target domain representations, we introduce a domain alignment loss that suppresses high similarity between different class responses within the same target sample prediction. Inspired by consistency-based learning strategies from MIC ^9^, this loss encourages structured separation in the predicted class distribution.

Given a target-domain input $x^t$, the model produces predicted probabilities $r_{\theta }(x^t) = [p_1, p_2, \dots , p_C]^\top \in \mathbb {R}^C$, where $p_j$ is the predicted probability of class $j$. Let $r_{\theta }(x^t)_j$ denote the response corresponding to class $j$. The domain alignment loss is defined as Eq. (2):

2 $$\begin{aligned} \mathscr {L}_{da}(x^t) = \frac{1}{C} \sum _{j=1}^{C} \sum _{\begin{array}{c} j'=1 \\ j' \ne j \end{array}}^{C} \left( \frac{r_{\theta }(x^t)_{j} \cdot \mathscr {U} \cdot r_{\theta }(x^t)_{j'}}{\Vert r_{\theta }(x^t)_{j}\Vert _2 \cdot \Vert r_{\theta }(x^t)_{j'}\Vert _2} \right) , \end{aligned}$$

where $\mathscr {U} \in \mathbb {R}^{C \times C}$ is a relational matrix that encodes interactions between different class responses. Minimizing this loss reduces the correlation between different classes in the predicted distribution of a target sample, thus improving class separation and domain alignment.

The discriminator plays an essential role in addressing the domain discrepancy through adversarial training. The adversarial loss function, presented in Eq. (3), distinguishes between source and target domain features:

3 $$\begin{aligned} \mathscr {L}_{adv}(x^s, x^t) = \mathbb {E}_{x_i^s \sim \mathscr {D}^s} [\log (d_{\epsilon }(f_i^s \oplus g_i^s))] + \mathbb {E}_{x_j^t \sim \mathscr {D}^t} [\log (1 - d_{\epsilon }(f_j^t \oplus g_j^t))], \end{aligned}$$

where $f_i^s, g_i^s$ and $f_j^t, g_j^t$ represent feature representations from source and target domain samples, respectively. The discriminator function $d_{\epsilon }$ with parameters $\epsilon$ classifies concatenated feature representations $f \oplus g$ as originating from the source or target domain. By maximizing the discriminator’s ability to differentiate between domains, the student network is driven to generate domain-invariant feature representations.

The overall optimization objective integrates supervised classification loss, domain alignment loss, and adversarial loss into a unified objective function as Eq. (4):

4 $$\begin{aligned} \min _{\varphi , \theta } \mathbb {E}_{(x_i^s, y_i^s) \sim \mathscr {D}^s} [\mathscr {L}_{sup}(x_i^s, y_i^s)] + \lambda _{da} \mathscr {L}_{da}(x^t) + \lambda _{adv} \mathscr {L}_{adv}(x^s, x^t), \end{aligned}$$

where $\lambda _{da}$ and $\lambda _{adv}$ are hyperparameters determining the relative contributions of the domain alignment and adversarial losses. The combination of these loss terms allows our model to effectively align feature representations across domains, facilitating robust knowledge transfer and improving model generalization on the unlabeled target domain.

### Multi-view affinity-based projection alignment

After feature extraction, the main purpose of data preprocessing is to make the feature spaces of the source and target domains consistent and reliable for subsequent model training and classification. To accomplish this, Principal Component Analysis (PCA) is used to reduce the dimensionality of the concatenated feature matrix $X$, which contains feature representations from both domains. Before PCA, the data is mean-centered by subtracting the mean vector $\mu$, computed as $\mu = \frac{1}{N} \sum _{i=1}^{N} X_i$. This ensures that the transformation reflects variance rather than absolute feature values. The centered data is then used to compute the covariance matrix $C$, followed by eigenvalue decomposition. The PCA transformation is formulated in Eq. (5):

5 $$\begin{aligned} X_{\text {reduced}} = (X - \mu ) P_{\text {pca}}, \quad \text {where} \quad P_{\text {pca}} = \arg \max _{P} \text {Tr}\bigl (P^T C P \bigr ), \quad C = \frac{1}{N} (X - \mu )^T (X - \mu ). \end{aligned}$$

Here, $P_{\text {pca}}$ consists of the top $k$ eigenvectors associated with the largest eigenvalues of $C$, capturing directions of maximum variance in the data. This transformation maintains most of the data variance in fewer dimensions, improving computational efficiency and promoting the alignment of source and target domain features. Once dimensionality is reduced, the features are normalized so that their L2 norm equals 1, as specified in Eq. (6):

6 $$\begin{aligned} \tilde{x}_i = \frac{x_i}{\Vert x_i\Vert _2}, \quad \forall x_i \in X_{\text {reduced}}, \end{aligned}$$

where $\tilde{x}_i$ denotes the normalized feature and $\Vert \cdot \Vert _2$ represents the L2 norm. The normalized features from both the source and target domains are then set to an equal scale, preparing them for classification and adversarial training.

After dimensionality reduction and normalization, the source and target features are merged to allow the model to learn shared and domain-specific information. This merged representation is key for cross-domain learning since the model can simultaneously process the joint features for domain alignment and classification.

Next, affinity matrices are built to capture label and feature relationships across the source and target domains. By fusing label-based and feature-based similarities, local and global structure is jointly considered. Specifically, the final affinity matrix $W_{\text {all}}$ is formed by combining label consistency and feature similarity with a weighting coefficient $\alpha$. If two samples share the same known label, their similarity is increased by adding $\alpha$. Otherwise, their similarity is determined by the Gaussian kernel distance. Eq. (7) defines $W_{\text {all}}$:

7 $$\begin{aligned} W_{\text {all}}(i, j) = {\left\{ \begin{array}{ll} \alpha + (1 - \alpha ) \exp \Bigl (-\frac{\Vert \textbf{x}_S(i) - \textbf{x}_T(j)\Vert ^2}{2\sigma ^2}\Bigr ), & \text {if } y_S(i) = y_T(j) \text { and } y_S(i) > 0, \\ (1 - \alpha ) \exp \Bigl (-\frac{\Vert \textbf{x}_S(i) - \textbf{x}_T(j)\Vert ^2}{2\sigma ^2}\Bigr ), & \text {otherwise}, \\ 0, & \text {if } i = j. \end{array}\right. } \end{aligned}$$

By adjusting $\alpha$, one can modulate the importance of label-based and feature-based similarity, creating a robust representation for the subsequent LPP and domain alignment steps. To enforce local consistency, the Laplacian matrix $L$ is derived from $W_{\text {all}}$ in Eq. (8):

8 $$\begin{aligned} L = D - W_{\text {all}}, \end{aligned}$$

where $D$ is a diagonal matrix whose entries are the row-sums of $W_{\text {all}}$. LPP then aims to minimize the projection loss $\text {argmin}_P \bigl (P^T L P\bigr )$ via eigenvalue decomposition of $L$. The eigenvectors associated with the smallest eigenvalues form the projection matrix $P$, which projects data into a lower-dimensional space while preserving local relationships.

After learning the projection matrix $P$, source and target features are projected into this shared lower-dimensional space according to Eq. (9):

9 $$\begin{aligned} \tilde{X}_{\text {proj}} = X_{\text {concat}} P, \quad \text {where} \quad \tilde{X}_{\text {proj}} = {\left\{ \begin{array}{ll} \tilde{X}^s_{\text {proj}}, & X_{\text {concat}} = X^s_{\text {concat}}, \\ \tilde{X}^t_{\text {proj}}, & X_{\text {concat}} = X^t_{\text {concat}}. \end{array}\right. } \end{aligned}$$

This unified representation allows one to measure how well target features align with the source distribution. The class mean distance is computed between the projected target features and the source class mean $\mu _S$. Similarly, cluster mean distance is computed between the projected target features and the target cluster mean $\mu _T$, ensuring that the target domain retains its internal structure. Meanwhile, minimizing these distances aids in aligning source and target features. A pseudo-label refinement mechanism updates target pseudo-labels using these distances and the label confidence, represented by Eq. (10):

10 $$\begin{aligned} \tau _T(i) = \frac{ \exp \Bigl ( - \frac{\Vert \textbf{x}_T^{\text {proj}}(i) - \mu _S \Vert ^2}{\sigma _1^2} \Bigr ) }{ \exp \Bigl ( - \frac{\Vert \textbf{x}_T^{\text {proj}}(i) - \mu _T \Vert ^2}{\sigma _2^2} \Bigr ) + \exp \Bigl ( - \frac{\Vert \textbf{x}_T^{\text {proj}}(i) - \mu _S \Vert ^2}{\sigma _1^2} \Bigr ) }, \end{aligned}$$

where $\sigma _1$ and $\sigma _2$ determine how strongly distances to source and target means influence label confidence. If the predicted confidence score of a sample is relatively low within its predicted class, the pseudo-label is considered unreliable and removed, as shown in Eq. (11):

11 $$\begin{aligned} \text {pseudoLabels}_T(i) = {\left\{ \begin{array}{ll} \text {predLabels}_T(i), & \text {if } \text {prob}(i) > \theta _{c(i)} \\ -1, & \text {otherwise} \end{array}\right. } \end{aligned}$$

Here, $\text {prob}(i)$ denotes the maximum class probability for the $i$-th target sample, and $\theta _{c(i)}$ is a class-specific threshold determined by the $(1 - p)$-quantile of predicted probabilities within class $c(i)$. The parameter $p = 1 - \frac{\text {iter}}{N}$ decreases linearly with the number of training iterations, where $\text {iter}$ is the current iteration and $N$ is the total number of iterations.

This class-aware pseudo-label selection strategy dynamically filters out unreliable target samples based on intra-class confidence statistics. As training progresses and the model’s prediction reliability improves, the selection threshold relaxes, enabling more pseudo-labeled target samples to be included. These high-confidence samples are then incorporated into the affinity graph by updating the similarity matrix $W$, where label similarity is weighted by a fixed coefficient $\alpha$.

Although $\alpha$ remains constant, its effective impact increases during training due to the growing number of trusted pseudo-labels included in the graph. This implicitly enhances label-based similarity and improves alignment quality over time. Such a confidence-guided graph construction not only mitigates error propagation but also improves the robustness and adaptability of cross-domain representation learning.

Additionally, a multi-view mechanism aggregates the predictions from multiple views by generating a probability matrix $\textbf{P}_v$ per view ($v=1, 2, 3$) and merging them into a fused probability matrix $\textbf{P}_{\text {fused}}$. The weight $\alpha _v$ for each view is defined via a distance-based Gaussian function, as in Eq. (12):

12 $$\begin{aligned} \textbf{P}_{\text {fused}} = \sum _{v=1}^{3} \alpha _v \cdot \textbf{P}_v, \quad \alpha _v = \frac{ \exp \Bigl ( - \frac{D_v^2}{\gamma _v^2} \Bigr ) }{ \sum _{v=1}^{3} \exp \Bigl ( - \frac{D_v^2}{\gamma _v^2} \Bigr ) }. \end{aligned}$$

Here, $\textbf{P}_v(i)$ is the softmax probability of the $i$-th target sample in view $v$. The final label is obtained by selecting the class with the highest probability as in Eq. (13):

13 $$\begin{aligned} \hat{y}_T(i) = \arg \max _j \bigl ( \textbf{P}_{\text {fused}}(i, j) \bigr ), \quad \forall i \in \{1, \dots , N_T\}. \end{aligned}$$

Algorithm 1 shows the procedure of MAPA. It first projects the source and target features with PCA and normalizes them. It then constructs a comprehensive affinity matrix, applies LPP to preserve local structures, updates target pseudo-labels based on source and target mean distances, and fuses multiple predicted probability matrices. Through dynamic weighting and confidence-based label refinement, the model improves alignment and classification accuracy in a shared lower-dimensional space.

**Algorithm 1:**
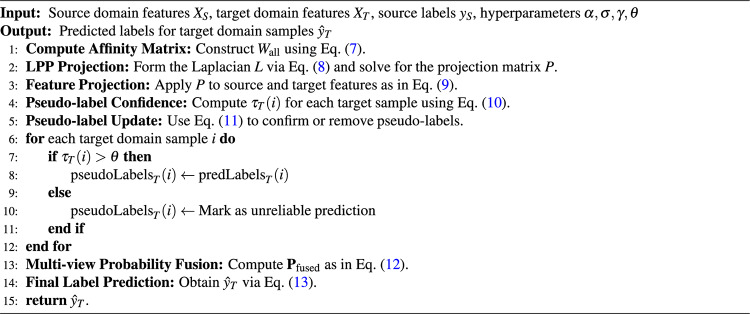
Multi-view Affinity-based Projection Alignment

## Experimental results and analysis

### Datasets and experimental setup

To validate the effectiveness of the proposed method, we conduct comprehensive experiments on three widely used benchmarks in UDA: ImageCLEF-DA, Office-Home, and VisDA-2017. These datasets present varying levels and types of domain shifts, enabling a rigorous and diverse evaluation of the proposed approach (Table 2).

**Table 2:** Training hyperparameters and their configurations.

Parameter	Description	Value
Iterations	Number of training epochs	10
Block size	Parallel processing unit size	1000
Projection dim	Dimensionality of the projected feature space	64/128
$\alpha$	Coefficient for label-based weighting	0.5
$\sigma$	Bandwidth of the RBF kernel	1.0

#### Benchmarks for evaluation

We utilize the following datasets for empirical evaluation:

ImageCLEF-DA^44^ This benchmark contains 12 shared object categories across three visual domains: Caltech-256 (C), ImageNet ILSVRC 2012 (I), and Pascal VOC 2012 (P). It defines six domain adaptation tasks, e.g., C $\rightarrow$ I, I $\rightarrow$ P, which serve to assess the model’s ability to generalize across related but distinct distributions.

Office-Home^45^ Office-Home comprises approximately 15,500 images belonging to 65 object categories, distributed across four distinct domains: Artistic (Ar), Clipart (Cl), Product (Pr), and Real-World (Rw). A total of twelve domain adaptation tasks, e.g., Ar $\rightarrow$ Cl, Pr $\rightarrow$ Rw, are defined, allowing for detailed evaluation of adaptation under complex domain shifts.

VisDA-2017^46^ This large-scale dataset focuses on the challenging synthetic-to-real adaptation scenario. It comprises 12 object categories spanning synthetic and real domains, specifically rendered 3D objects and photographic images. The pronounced domain discrepancy makes this benchmark especially suitable for evaluating the robustness of UDA methods.

#### Implementation and experimental configuration

We implement our method using PyTorch for deep feature extraction and MATLAB for LPP and affinity matrix computation. To ensure a fair comparison with existing approaches, we adopt multiple backbone architectures depending on the dataset:

- ResNet-50 (2048-dimensional output) for Office-Home and ImageCLEF-DA.
- ResNet-101 (2048-dimensional output) for VisDA-2017.
- ViT (768-dimensional output) for cross-architecture evaluations.

The hyperparameters were carefully selected through empirical tuning to achieve optimal performance across different datasets. The projection dimensionality was set to 64 for Office-Home and 128 for ImageCLEF-DA and VisDA-2017 to balance feature compactness and discriminative power. Training iterations were limited to 10 to ensure computational efficiency while maintaining convergence. We employed a fixed block size of 1000 samples per batch to optimally balance computational demands with topological structure preservation. For the label weighting, we used a coefficient of 0.5 to equally weigh feature similarity and pseudo-label guidance. Finally, the RBF kernel bandwidth was configured as 1.0 to enable the effective transformation of feature distances into Gaussian similarity measures while preserving local data structures. This comprehensive parameter configuration ensures robust performance while maintaining computational efficiency across different experimental settings.

### Empirical evaluation and comparative analysis

**Table 3:** Classification accuracy (%) on the ImageCLEF-DA dataset for various domain adaptation methods. The best results are highlighted in bold.

Method	I$\rightarrow$P	P$\rightarrow$I	I$\rightarrow$C	C$\rightarrow$I	C$\rightarrow$P	P$\rightarrow$C	Avg.
Baseline	74.8	83.9	91.5	78.0	65.5	91.2	80.82
CDAN ^19^	77.7	90.7	97.7	91.3	74.2	94.3	87.65
ASAN ^35^	78.9	92.3	97.4	92.1	76.4	94.4	88.58
CGDM ^21^	78.7	93.3	97.5	92.7	79.2	95.7	89.52
GLOT-DR ^40^	**81.0**	91.7	97.9	93.3	79.5	95.0	89.73
SPL ^8^	78.3	**94.5**	96.7	**95.7**	**80.5**	96.3	90.33
Ours	80.0	94.4	**98.1**	94.3	79.2	**97.2**	**90.54**

**Table 4:** Comparison of domain adaptation methods on the Office-Home dataset using ResNet-50 and ViT backbones. The best results for each task are highlighted in bold.

Method	Backbone	A$\rightarrow$C	A$\rightarrow$P	A$\rightarrow$R	C$\rightarrow$A	C$\rightarrow$P	C$\rightarrow$R	P$\rightarrow$A	P$\rightarrow$C	P$\rightarrow$R	R$\rightarrow$A	R$\rightarrow$C	R$\rightarrow$P	AVG
Baseline	ResNet-50	34.9	50.0	58.0	37.4	41.9	46.2	38.5	31.2	60.4	53.9	41.2	59.9	46.13
CDAN ^19^		50.7	70.6	76.0	57.6	70.0	70.0	57.4	50.9	77.3	70.9	56.7	81.6	65.81
ASAN ^35^		53.6	73.0	77.0	62.1	73.9	72.6	61.6	52.8	79.8	73.3	60.2	83.6	68.63
SPL ^8^		54.5	77.8	81.9	65.1	78.0	81.1	66.0	53.1	82.8	69.9	55.3	86.0	70.96
BIWAA ^20^		56.3	78.4	81.2	68.0	74.5	75.7	67.9	56.1	81.2	75.2	60.1	83.8	71.53
CLIP ^14^		51.6	**81.9**	82.6	**71.9**	**81.9**	**82.6**	**71.9**	51.6	82.6	71.9	51.6	81.9	72.00
SDAT ^39^		58.2	77.1	82.2	66.3	77.6	76.8	63.3	57.0	82.2	74.9	64.7	86.0	72.19
ELS		58.2	79.7	82.5	67.5	77.2	77.2	64.6	57.9	82.2	75.4	63.1	85.5	72.58
CoVi ^22^		58.5	78.1	80.0	68.1	80.0	77.0	66.4	**60.2**	82.1	**76.6**	63.6	**86.5**	73.09
GSDE ^37^		57.8	80.2	81.9	71.3	78.9	80.5	67.4	57.2	**84.0**	76.1	62.5	85.7	73.63
Ours		**63.2**	79.9	**83.5**	68.5	74.7	79.4	67.6	58.4	83.0	76.5	**65.6**	86.1	**73.86**
Baseline	ViT	66.2	84.3	86.6	77.9	83.3	84.3	76.0	62.7	88.7	80.1	66.2	88.7	78.74
CLIP ^14^		67.8	89.0	89.8	82.9	89.0	89.8	82.9	67.8	89.8	82.9	67.8	89.0	82.38
TVT ^24^		74.9	86.8	89.5	82.8	88.0	88.3	79.8	71.9	90.1	85.5	74.6	90.6	83.56
SDAT ^39^		70.8	87.0	90.5	85.2	87.3	89.7	84.1	70.7	90.6	88.3	75.5	92.1	84.32
ELS ^38^		72.1	87.3	90.6	85.2	88.1	89.7	84.1	70.7	90.8	88.4	76.5	92.1	84.63
PGA ^36^		71.8	91.5	91.0	84.8	**91.6**	90.9	**84.9**	71.5	91.1	85.9	72.1	92.4	84.96
MIC ^9^		80.2	87.3	91.1	87.2	90.0	90.1	83.4	75.6	**91.2**	**88.6**	78.7	91.4	86.23
Ours		**80.7**	**91.6**	**91.9**	**87.9**	90.0	**91.2**	84.6	**76.4**	91.0	88.3	**79.9**	**93.1**	**87.21**

**Table 5:** Classification accuracy (%) on the VisDA-2017 dataset for various domain adaptation methods. The best results are highlighted in bold.

Method	Backbone	airplane	bicycle	bus	car	horse	knife	motorcycle	person	plant	skateboard	train	truck	AVG
Baseline	ResNet-101	55.1	53.3	61.9	59.1	80.6	17.9	79.7	31.2	81.0	26.5	73.5	8.5	52.36
CGDM ^21^		93.4	82.7	73.2	68.4	92.9	94.5	88.7	82.1	93.4	82.5	86.8	49.2	82.32
SDAT ^39^		95.8	85.5	76.9	69.0	93.5	97.4	88.5	78.2	93.1	91.6	86.3	55.3	84.26
CLIP ^14^		**98.2**	**83.9**	**90.5**	73.5	**97.2**	84.0	**95.3**	65.7	79.4	89.9	**91.8**	**63.3**	84.39
Ours		96.7	**83.9**	80.9	**76.2**	97.0	**98.1**	90.0	**84.0**	**96.5**	**94.7**	88.7	57.5	**87.01**
Baseline	ViT	98.2	73.0	82.5	62.0	97.3	63.5	96.5	29.8	68.7	86.7	96.7	23.7	73.22
TVT ^24^		92.9	85.6	77.5	60.5	93.6	98.2	89.4	76.4	93.6	92.0	91.7	55.7	83.92
PMTrans ^23^		98.9	93.7	84.5	73.3	99.0	98.0	96.2	67.8	94.2	**98.4**	96.6	49.0	87.47
CLIP ^14^		99.3	91.7	**93.9**	74.3	98.4	94.3	90.3	78.2	78.3	97.3	95.2	64.8	88.00
SDAT ^39^		98.4	90.9	85.4	82.1	98.5	97.6	96.3	86.1	96.2	96.7	92.9	56.8	89.83
CMKD ^13^		**99.4**	94.6	91.5	78.9	98.7	97.3	93.3	81.3	91.8	97.9	**96.9**	61.7	90.28
Ours		98.4	**94.8**	87.2	**88.7**	**100.0**	**100.0**	**97.6**	**89.0**	**97.8**	98.3	92.0	**68.5**	**92.68**

Table 3 presents classification results for various domain adaptation techniques on the ImageCLEF-DA benchmark. The baseline model achieves a mean accuracy of 80.82%, reflecting limited capability in addressing complex domain shifts. CDAN boosts the performance to 87.65%, notably improving I$\rightarrow$C and C$\rightarrow$I through conditional adversarial alignment. ASAN attains 88.58%, showing enhancements in P$\rightarrow$I and C$\rightarrow$I by leveraging adaptive sample alignment.

CGDM reaches 89.52%, benefiting from category-level dynamic matching, especially in C$\rightarrow$P. GLOT-DR slightly improves the results to 89.73%, achieving the best I$\rightarrow$P accuracy due to its global-local feature regularization. SPL obtains 90.33%, achieving the highest performance on three tasks via progressive self-paced learning.

Our proposed method surpasses all existing approaches with an average accuracy of 90.54%. It delivers state-of-the-art performance on I$\rightarrow$C with 98.1% accuracy and P$\rightarrow$C with 97.2% accuracy, and remains second-best across the remaining tasks, indicating robust cross-domain alignment, especially in high-modality-gap scenarios.

These results confirm the effectiveness of the MAPA framework in capturing both semantic and structural domain information. The model generalizes well across diverse domain pairs, especially in challenging transitions such as between clipart and product images.

Table 4 shows the classification performance of domain adaptation methods on the Office-Home dataset using both ResNet-50 and ViT architectures. With ResNet-50, the baseline yields 46.13% accuracy, indicating limited adaptation ability. Methods including CDAN with 65.81% accuracy and ASAN with 68.63% accuracy demonstrate consistent improvements through feature alignment strategies. More advanced approaches, such as SPL achieving 70.96% and BIWAA reaching 71.53% further enhance performance by emphasizing task-relevant features.

CLIP and CoVi achieve superior performance with 72.00% and 73.09% accuracy, respectively, benefiting from pre-training and contrastive learning approaches. Our method achieves 73.86%, the best among all, showing effective integration of label smoothing, affinity-guided projection, and multi-view learning.

With ViT, accuracy improves significantly across methods. The baseline reaches 78.74%, while CLIP, TVT, and SDAT push it above 82%. MIC and PGA achieve 86.23% and 84.96%, respectively. Our method achieves the highest accuracy of 87.21%, confirming its robustness on transformer-based models.

Our method ranks first in 3 ResNet-50 tasks and in 8 ViT tasks, showing consistent dominance or competitive performance across all pairs. Tasks such as A$\rightarrow$C and C$\rightarrow$A remain difficult due to domain gaps, and future work should target them specifically.

Table 5 presents the classification accuracy on the VisDA-2017 dataset, comparing our method against several domain adaptation approaches using both ResNet-101 and ViT backbones.

With the ResNet-101 backbone, the baseline achieves only 52.36% average accuracy, revealing the limitations of standard convolutional networks in high-shift domain scenarios. CGDM, SDAT, and CLIP improve performance significantly, reaching 82.32%, 84.26%, and 84.39% respectively, by leveraging category-level matching, adversarial training, and vision-language pretraining. Our method achieves the highest average accuracy of 87.01%, with particularly strong performance in challenging categories: knife at 98.1%, person at 84.0%, and plant at 96.5% accuracy, surpassing all competing approaches.

With the ViT backbone, performance improves across all models. The baseline achieves 73.22% accuracy, while transformer-based methods show consistent improvements: PMTrans reaches 87.47%, SDAT attains 89.83%, and CMKD achieves 90.28%. Our method establishes new state-of-the-art performance with 92.68% accuracy, demonstrating superior results across all categories. Notably, the method achieves perfect 100.0% accuracy for both horse and knife recognition, along with 97.8% accuracy for plant classification. It also maintains strong performance in challenging categories, reaching 88.7% for car and 68.5% for truck recognition, surpassing all existing approaches.

These results confirm that transformer-based models extract more generalizable features and that our proposed strategy effectively bridges domain gaps. The ViT-based variant of our method consistently ranks first or second across all categories, showing high stability and robustness.

Difficult categories, such as truck and person, display high intra-class variability and present greater adaptation challenges. Future efforts will aim to enhance performance in these categories by introducing finer-grained alignment techniques and stronger regularization. Enhancing domain invariance through adaptive attention, robust curriculum strategies, and dynamic feature mixing will be explored.

Overall, our method sets a new benchmark on VisDA-2017 across both backbones, indicating the general applicability of our approach to large-scale, category-diverse domain adaptation tasks.

Although the proposed MAPA framework demonstrates competitive overall performance, specific object categories such as truck and person in the VisDA-2017 dataset remain challenging. This can be attributed to significant domain-induced variations in shape, scale, and appearance across these categories, which lead to increased intra-class diversity and inter-class confusion. To mitigate this, future extensions of MAPA could incorporate class-aware reweighting strategies to focus training on underperforming classes. Additionally, integrating fine-grained attention mechanisms or category-specific feature disentanglement modules may help isolate domain-invariant semantic cues, thereby enhancing class-level alignment. Another promising direction is to apply targeted augmentation or pseudo-label calibration techniques based on class-wise confidence statistics to refine supervision signals for difficult categories.

**Fig. 2:**
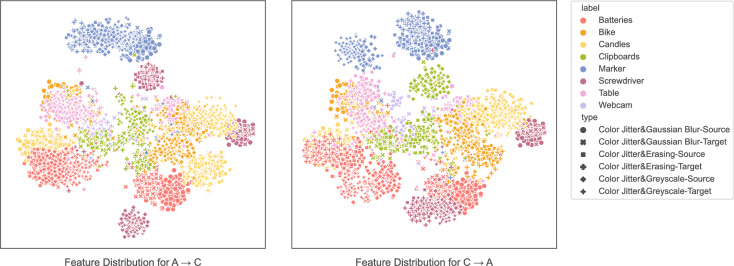
t-SNE visualization of feature distributions for two challenging domain adaptation tasks: A$\rightarrow$C and C$\rightarrow$A .

**Fig. 3:**
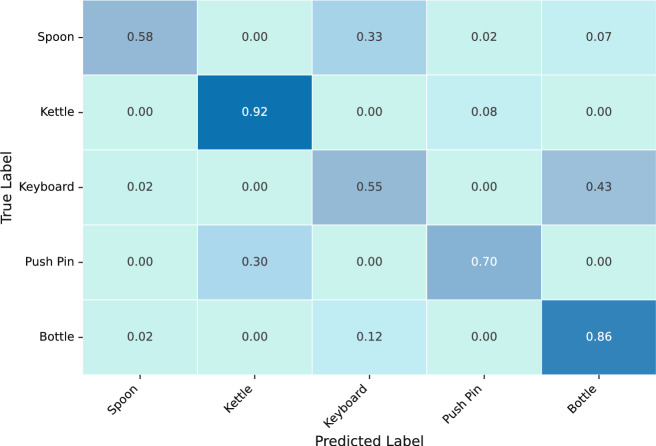
Confusion matrix for the A$\rightarrow$C task on the Office-Home dataset. The matrix highlights common misclassification patterns between semantically similar categories, such as Keyboard and Bottle.

### Analysis on inter-domain heterogeneity

To investigate the notably lower performance observed in the A$\rightarrow$C and C$\rightarrow$A tasks reported in Table 4, we conduct a thorough analysis based on feature distribution visualization and confusion matrix inspection.

Figure 2 presents the t-SNE visualization of feature representations learned by our model on the A$\rightarrow$C and C$\rightarrow$A tasks. In the A$\rightarrow$C task, we observe substantial overlap between semantically different classes and a clear misalignment between source and target domain samples. For example, categories such as Bike, Candles, and Table form entangled clusters, and features of the same class from different domains are often split into subclusters, indicating a lack of domain-invariance. For the C$\rightarrow$A task, the intra-class compactness is relatively improved, but domain discrepancy persists, especially for samples affected by strong augmentations such as color jitter and grayscale transformation. These results confirm that domain-specific characteristics and visual abstraction in the Clipart and Amazon domains introduce structural heterogeneity that complicates alignment.

To further investigate the performance degradation in the A$\rightarrow$C task, we analyze the confusion matrix shown in Fig. 3. While categories such as Kettle and Bottle achieve relatively high prediction accuracy (92% and 86%, respectively), other classes suffer from severe misclassification. For instance, only 58% of Spoon samples are correctly classified, while 33% are incorrectly predicted as Keyboard. Similarly, 43% of Keyboard instances are confused with Bottle, resulting in substantial category overlap. This confusion can be attributed to visual similarities among classes under the Clipart domain, where stylized abstraction weakens edge details and shape contours. Spoon, Keyboard, and Bottle share elongated and narrow shapes, which makes it challenging for the model to distinguish between them without rich texture cues. In addition, 30% of Push Pin samples are misclassified as Kettle, likely due to the presence of rounded tops and small object size in both categories, leading to semantic ambiguity in stylized representations.

In summary, the degradation in performance for these tasks is attributed to both domain-level structural misalignment and class-level visual similarity. Future improvements may focus on class-aware domain alignment and adaptive augmentation to enhance model robustness in challenging transfer scenarios.

### Effect of LPP dimensionality on unsupervised domain adaptation performance

**Fig. 4:**
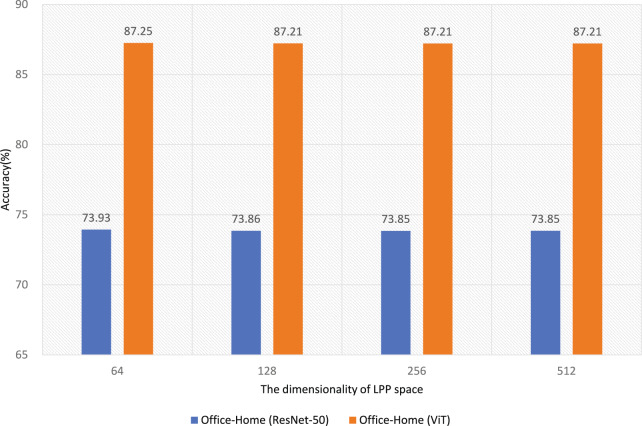
Impact of LPP dimensionality on domain adaptation performance for the Office-Home dataset using ResNet-50 and ViT backbones.

Figure 4 presents the results of evaluating the effect of LPP dimensionality on unsupervised domain adaptation performance for the Office-Home dataset using ResNet-50 and ViT backbones. The experimental results indicate that LPP dimensionality has negligible influence on classification accuracy for both architectures.

For the ResNet-50 backbone, classification accuracy shows consistent stability across all tested LPP dimensions, including 64, 128, 256, and 512, with accuracy values ranging narrowly from 73.93% to 73.85%. This minimal variation suggests that increasing the LPP dimension does not significantly contribute to feature alignment or classification improvement. A similar trend is observed for the ViT backbone, where accuracy fluctuates slightly between 87.25% and 87.21%. These findings imply that essential discriminative information is preserved even in lower-dimensional LPP spaces and that higher-dimensional projections do not offer tangible performance gains.

Notably, increasing the LPP dimensionality from 64 to 128 does not result in meaningful improvements, reinforcing the conclusion that lower-dimensional projections are sufficient. Beyond 128 dimensions, further increases introduce no observable benefit, indicating potential redundancy in the added dimensions.

ViT consistently achieves higher accuracy than ResNet-50 at all LPP dimensionalities, with an average margin of approximately 13 percentage points. This performance gap reflects ViT’s capability to capture global contextual features, which facilitates more effective cross-domain alignment. In contrast, ResNet-50’s local feature representations appear less adaptable to domain shift, which may explain the limited sensitivity of its performance to LPP dimensionality.

In summary, the dimensionality of the LPP space does not significantly impact domain adaptation performance. Lower-dimensional projections, such as 64 or 128 dimensions are adequate for maintaining discriminative features. Furthermore, ViT’s consistent outperformance across all LPP dimensions underscores the effectiveness of global feature modeling in enhancing adaptation performance.

### Sensitivity analysis of hyperparameters

To assess the robustness of our framework with respect to the hyperparameter $\alpha$, which controls the weighting between label-based and feature-based similarity in the affinity matrix construction, we conduct a sensitivity analysis on two representative benchmarks: Office-Home and ImageCLEF-DA, both using ResNet-50 as the backbone. The parameter $\alpha$ is varied from 0 to 1 with a step size of 0.1, and the corresponding classification accuracy is recorded.

**Fig. 5:**
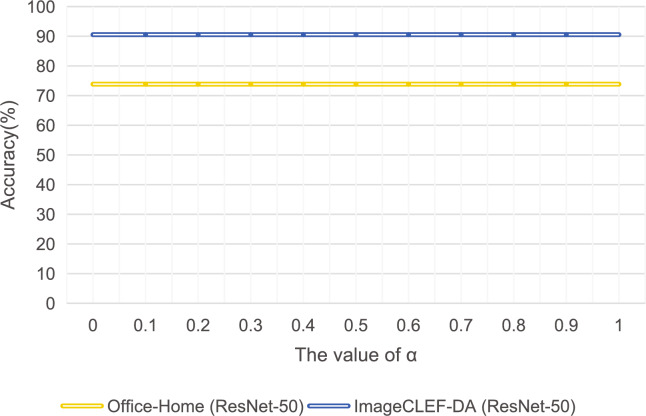
Accuracy comparison under different values of $\alpha$ on Office-Home and ImageCLEF-DA datasets.

As shown in Fig. 5, the accuracy on the Office-Home dataset remains consistently at 73.86% across all tested values of $\alpha$. Likewise, the accuracy on ImageCLEF-DA also remains unchanged at 90.54%. This remarkable invariance suggests that our method is highly insensitive to the selection of $\alpha$, demonstrating strong robustness of the proposed affinity fusion mechanism.

The observed stability indicates that the model effectively leverages both label and feature information, regardless of their relative weighting. Even when relying solely on feature similarity or label consistency, the framework maintains stable performance. This robustness can be attributed to the joint optimization of pseudo-label refinement and multi-view fusion, which compensates for potential imbalances introduced by varying $\alpha$.

Overall, the experimental results confirm that $\alpha$ does not significantly influence the performance of our model. This insensitivity enhances the practical utility of the proposed framework by reducing the reliance on careful hyperparameter tuning. Despite this robustness, future work may explore adaptive parameter selection strategies that dynamically adjust $\alpha$ based on the estimated intensity of domain shifts. By incorporating domain shift indicators or statistical divergence measures, such methods could provide a more principled and interpretable way to balance label-based and feature-based affinities, especially in open-world or non-stationary adaptation scenarios.

### Ablation study

To evaluate the effectiveness of the affinity matrix in our MGPA framework, we conduct an ablation study by replacing the complete graph-based processing pipeline with a conventional K-Nearest Neighbor (KNN) classifier. This modification specifically removes the graph construction module that generates affinity scores through combined feature similarity and label consistency metrics, along with the structured projection module employing Laplacian regularization for subspace learning. In this simplified version, target samples are classified through direct Euclidean nearest-neighbor matching - each target instance is paired with its closest source sample based on Euclidean distance, with the source label directly transferred without feature space projection. This controlled substitution isolates the impact of graph-based processing while maintaining identical feature extraction and classification components, ensuring a valid comparison of domain adaptation strategies.

**Table 6:** Effect of the affinity matrix on domain adaptation accuracy on the Office-Home dataset.

Method	ResNet-50	ViT
k-NN (Euclidean)	70.17	82.78
+ Affinity Matrix	73.86 ($\uparrow$3.69)	87.21 ($\uparrow$4.43)

**Table 7:** Effect of PCA and LPP on domain adaptation accuracy using ResNet-50 and ViT on the Office-Home dataset.

Method	Office-Home (ResNet-50)	Office-Home (ViT)
PCA Only	73.73	87.19
LPP Only	73.50	87.09
PCA + LPP	73.86	87.21

As demonstrated in Table 6, the substitution results in significant performance degradation. With the ResNet-50 backbone, the accuracy drops from 73.86% achieved by our affinity matrix-enhanced method to merely 70.17% for the KNN baseline, indicating a substantial improvement of 3.69 percentage points. The performance gap becomes even more pronounced when employing the ViT backbone, where our approach reaches 87.21% accuracy compared to 82.78% for KNN, demonstrating a notable 4.43 percentage point advantage. These consistent performance differentials reveal the inherent limitations of the KNN-based mechanism, which relies solely on local pairwise distance computations and lacks both structural modeling capacity and transductive inference capability. In contrast, the affinity matrix in MGPA builds a global similarity graph that combines semantic similarity from pseudo-labels and structural similarity from feature distances. This graph guides the construction of a locality-preserving projection subspace that captures both global alignment and local geometric consistency. The resulting representation allows the model to more effectively align source and target distributions, while also enabling more reliable pseudo-label refinement through the iterative updating steps in our algorithm. Notably, the larger performance gain observed with the ViT backbone suggests that its self-attention mechanism benefits more from the global structural priors encoded in the graph, reinforcing the synergy between graph-based alignment and transformer-based feature extraction.

We further analyze the effect of feature transformation using PCA and LPP for domain adaptation by designing an ablation study with three configurations: PCA only, LPP only, and their combination on the Office-Home dataset. In the complete MGPA framework, the source and target features are first transformed via PCA for dimensionality reduction and then projected using LPP to preserve the local geometric structure. To examine the role of each component individually, we make targeted modifications to the algorithm. In the “PCA only” setting, the LPP projection step is removed, and classification is performed directly on the features after PCA transformation, without learning a locality-preserving subspace. Conversely, in the “LPP only” setting, the PCA step is omitted, and the affinity matrix as well as LPP are applied directly to the original high-dimensional features. This configuration isolates the contribution of the structure-preserving projection alone. The third configuration retains both PCA and LPP steps as in the original pipeline.

As evidenced in Table 7, PCA alone yields strong performance with 73.73% accuracy for ResNet-50 and 87.19% for ViT, indicating its effectiveness in capturing the most discriminative directions while reducing noise. LPP alone performs slightly worse, achieving 73.50% and 87.09% respectively, likely because it emphasizes local neighborhood preservation but does not retain global variance effectively. When both PCA and LPP are applied sequentially, accuracy improves further to 73.86% for ResNet-50 and 87.21% for ViT. This improvement suggests that PCA reduces data dimensionality and noise, providing a more compact representation for LPP to focus on local geometric structures. The results demonstrate that PCA and LPP are complementary in nature: PCA captures global patterns, while LPP preserves local relationships. Their combination improves feature alignment and boosts domain adaptation performance across both backbone types.

**Table 8:** Impact of different data augmentation strategies on domain adaptation performance across datasets and backbones.

Gaussian blur	Erasing	Grayscale	ImageCLEF	Office-Home (ResNet-50)	Office-Home (ViT)	VisDA-2017 (ResNet-101)
$\checkmark$			91.03	70.81	86.37	87.80
	$\checkmark$		90.37	71.40	86.38	87.20
		$\checkmark$	90.36	71.44	86.35	86.25
$\checkmark$	$\checkmark$		90.75	71.57	86.46	84.53
$\checkmark$		$\checkmark$	90.19	71.95	86.49	84.68
	$\checkmark$	$\checkmark$	89.84	72.29	86.63	84.19
$\checkmark$	$\checkmark$	$\checkmark$	90.53	73.86	87.21	87.01

**Fig. 6:**
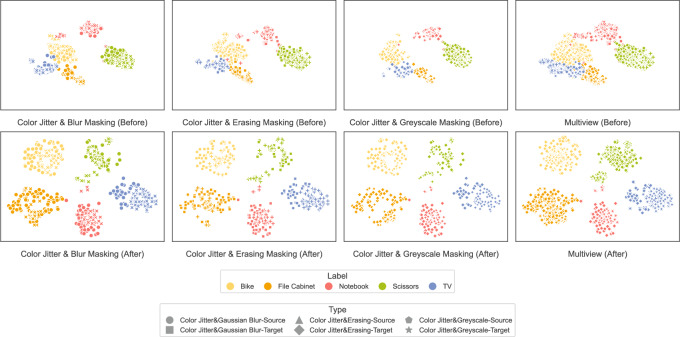
t-SNE visualization of feature distributions in the Office-Home dataset using ResNet-50. The top row shows pre-adaptation distributions, and the bottom row shows post-adaptation distributions under multi-view augmentation.

To study the role of data augmentation in domain adaptation, we evaluate the individual and combined effects of Gaussian blur, random erasing, and grayscale transformations across multiple datasets and architectures. As detailed in Table 8, we report classification accuracies on ImageCLEF, Office-Home, and VisDA-2017 using three backbone networks: ResNet-50, ResNet-101, and ViT. These augmentations are applied during the feature extraction phase of our method, where each target sample is transformed into three distinct views, serving as inputs to the multi-view fusion module. In the ablation settings, we remove individual augmentation strategies to isolate their respective contributions. Specifically, each configuration corresponds to the removal of a single augmentation type from the multi-view stream, while keeping the rest of the MGPA pipeline unchanged. This allows us to assess how different views affect the quality of learned representations and the robustness of the final pseudo-label predictions.

Gaussian Blur yields the highest accuracy of 91.03% on ImageCLEF, indicating that global feature smoothing is beneficial in this case. However, its performance drops to 70.81% on Office-Home and 87.80% on VisDA-2017, likely due to the loss of fine-grained features. Random Erasing achieves improved performance with 71.40% accuracy on Office-Home and 87.20% on VisDA-2017, suggesting enhanced robustness through occlusion of discriminative regions. Grayscale transformation demonstrates competitive results at 71.44% accuracy on Office-Home but underperforms at 86.25% on VisDA-2017, implying that color removal helps in some cases but can be detrimental when color cues are essential.

Combinations of augmentations exhibit diverse behaviors. The Gaussian Blur with Random Erasing augmentation improves accuracy to 71.57% on Office-Home while reducing performance to 84.53% on VisDA-2017. Gaussian Blur with Grayscale achieves 71.95% in Office-Home and 84.68% in VisDA-2017, indicating its advantage in scenarios where color information is less crucial. The combination of Random Erasing and Grayscale reaches 72.29% on Office-Home, but its performance drops to 84.19% on VisDA-2017.

The best results are achieved when all three augmentations are applied jointly, reaching 73.86% on Office-Home and 87.01% on VisDA-2017. This demonstrates the effectiveness of multi-view augmentation in enhancing generalization by introducing diverse transformations that reduce domain-specific biases. For Office-Home with ResNet-50, this combination provides the most significant improvement, while ViT shows relatively stable performance across augmentation types, indicating its lower dependency on data augmentations due to its ability to capture global patterns.

To further investigate the impact of different augmentation strategies on cross-domain feature alignment, we visualize t-SNE projections of feature representations on the Office-Home dataset using ResNet-50 in Fig. 6. The top row shows the distribution before adaptation, and the bottom row displays the distribution after adaptation with multi-view augmentations.

Before adaptation, there is a clear separation between source and target domain features, with substantial misalignment. Categories such as File Cabinet and TV exhibit high variance in target features, indicating severe domain shift. In contrast, categories like Notebook and Scissors show better alignment, suggesting more robust shared representations.

After adaptation with various augmentation strategies, target samples are more closely aligned with source samples. Augmentation combinations lead to category-specific improvements. For example, Color Jitter with Gaussian Blur improves alignment in Bike and Scissors, likely due to the enhancement of shape-based features. Color Jitter with Erasing benefits Notebook and File Cabinet, suggesting that occlusion strengthens global context modeling. However, Color Jitter with Grayscale shows limited improvement for TV and File Cabinet, indicating that color information is essential for these categories.

Overall, the full combination of Gaussian Blur, Random Erasing, and Grayscale provides the most consistent improvements across all categories. These results confirm that multi-view augmentation enhances feature alignment by integrating diverse invariances. The combination of empirical and visual evidence supports the conclusion that multi-view augmentation significantly improves generalization in cross-domain settings.

### Effectiveness of multi-view augmentation on per-class adaptation

**Fig. 7:**
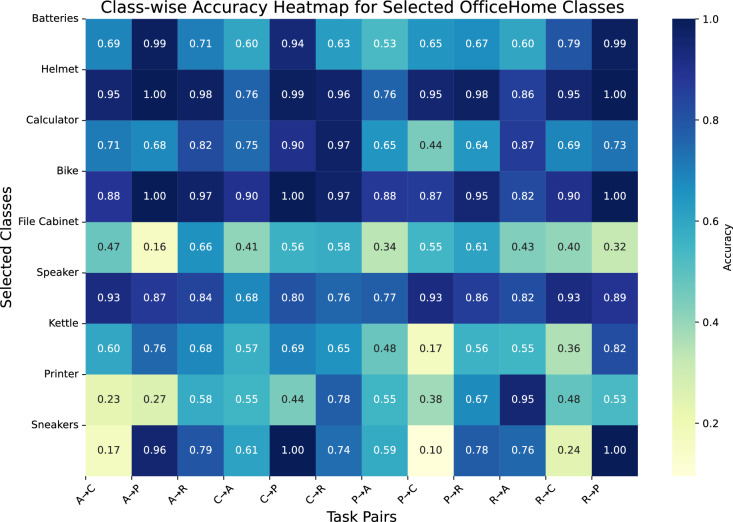
Class-wise accuracy heatmap on the Office-Home dataset (ResNet-50). Darker regions indicate higher classification accuracy. The heatmap highlights the influence of different augmentation strategies on individual object classes.

The class-wise accuracy analysis presented in Fig. 7 highlights the differential impact of data augmentation strategies on individual object categories in the Office-Home dataset. The analysis reveals that object classes exhibit varying levels of sensitivity to augmentation techniques, which can be attributed to differences in visual properties and contextual dependencies.

Object categories with distinctive geometric or structural features, such as helmets and bikes, consistently achieve high accuracy across all augmentation types. This indicates that these classes have discriminative features that are less affected by domain shift. In contrast, classes such as file cabinets and printers, which rely more heavily on contextual or background information, display substantial accuracy variability, suggesting that these objects are more susceptible to domain discrepancies.

Further examination of augmentation-specific effects provides insight into their individual contributions. Gaussian blur significantly improves the performance of edge-reliant objects, such as bikes, by enhancing the focus on global structural features. Random erasing yields superior results for context-sensitive categories like calculators, likely due to its capacity to encourage holistic feature learning by occluding partial regions. In contrast, grayscale transformation leads to a notable drop in accuracy for color-dependent classes like kettles, underscoring the risk of discarding essential chromatic cues during training.

**Fig. 8:**
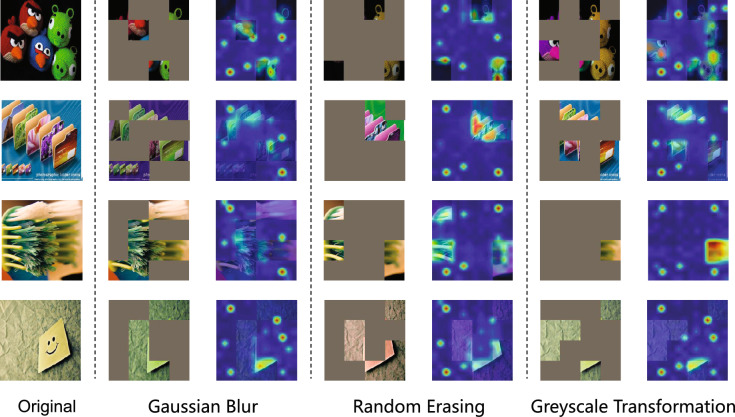
Attention map visualizations under different augmentation strategies on the Office-Home dataset (ResNet-50). Rows denote object categories: *Toys*, *Folder*, *Toothbrush*, and *Post-it Notes*. Columns correspond to augmentation techniques: Gaussian blur, Random erasing, and Grayscale transformation.

The proposed multi-view augmentation strategy, which integrates multiple augmentation types during training, demonstrates clear improvements over single-view methods. It not only preserves high accuracy for geometry-dominant categories but also significantly reduces performance variance across object types. For instance, the accuracy range for challenging categories such as file cabinets is substantially narrowed, indicating enhanced robustness. These results confirm that combining augmentations with complementary effects facilitates the learning of more transferable representations, which is critical for real-world domain adaptation scenarios involving diverse object categories.

To further assess how augmentation influences feature learning in unsupervised domain adaptation, we visualize attention maps generated by the ResNet-50 model trained with different augmentation strategies on the Office-Home dataset, as shown in Fig. 8. The visualizations provide qualitative evidence of how augmentations affect the model’s focus on object-relevant regions.

Each augmentation strategy induces distinct behavioral characteristics in the model. Gaussian blur promotes stronger reliance on structural contours rather than fine textures, as demonstrated by attention maps showing broad coverage of object shapes in categories such as toothbrushes and folders, indicating enhanced global spatial awareness. Random erasing increases tolerance to occlusions, with attention remaining focused on key regions despite partial information removal, particularly evident for objects like toys and Post-it notes. Grayscale transformation forces dependency on shape and edge features when color information is absent, resulting in more evenly distributed attention across object structures, as clearly observed in folder examples where geometric features dominate. These augmentation-specific responses collectively enhance the model’s robustness across diverse visual conditions.

These attention patterns are consistent with the quantitative performance improvements observed in the ablation studies, where combining all three augmentation strategies leads to the highest classification accuracy. The complementary nature of the augmentations—structural emphasis from Gaussian blur, occlusion resilience from random erasing, and reduced color dependency via grayscale transformation—collectively enhances the model’s ability to learn domain-invariant features. These results provide further justification for adopting multi-view augmentation in unsupervised domain adaptation tasks.

## Conclusion and future work

This paper presents a projection-based multi-view UDA framework that integrates label and feature similarities through a dynamically constructed affinity matrix. The proposed method employs LPP to map data from both source and target domains into a shared low-dimensional subspace, preserving local geometric structures. To enhance alignment, an adaptive pseudo-label refinement strategy is introduced, improving the reliability of target domain supervision. In addition, the framework incorporates multiple augmented views of the data, which improves feature robustness and mitigates the impact of domain shift. This multi-view fusion mechanism allows the model to capture complementary information across different feature spaces, thereby strengthening cross-domain generalization.

To maintain focus and interpretability, we selected three commonly used augmentations—Gaussian blur, random erasing, and grayscale conversion—for generating diverse feature views. These augmentations have demonstrated strong empirical performance in prior UDA and domain generalization studies. Nevertheless, we acknowledge that incorporating a broader range of augmentations such as color jittering and CutMix, or adopting an automated augmentation selection mechanism tailored to the source–target characteristics, could further enhance adaptability. We have included this consideration in the conclusion as a promising direction for future research.

Empirical evaluations on three widely used benchmarks demonstrate the effectiveness of the proposed approach across both convolutional neural network (CNN) and Transformer-based architectures. Our method consistently achieves higher accuracy compared to existing state-of-the-art techniques. Comprehensive ablation studies confirm the individual contributions of LPP, pseudo-label refinement, and multi-view fusion. Visualizations of the learned feature distributions further support the effectiveness of dynamic similarity weighting in aligning domains.

Future work may explore domain shifts involving multi-modal data, such as temporal or textual inputs, which may require new techniques to preserve local geometric structures in more complex representations. Moreover, given the promising results achieved with the ViT architecture, incorporating attention mechanisms from Transformers into CNN-based backbones could further enhance the framework’s adaptability. The integration of diffusion-based generative models also presents an exciting direction for improving domain alignment through synthetic data generation and more expressive feature representations. In addition, while the current work focuses on image classification, the proposed projection-based framework could potentially be extended to other tasks such as semantic segmentation, object detection, or cross-modal adaptation by adapting the affinity construction and label refinement components to suit task-specific requirements.

## Data Availability

The datasets generated and/or analysed during the current study are available in the Office-Home repository, https://www.hemanthdv.org/officeHomeDataset.html, VisDA-2017 repository, https://ai.bu.edu/visda-2017/ and ImageCLEF repository, https://www.imageclef.org/datasets.

## Code availability

The code will be made available after the manuscript is accepted.
